# Mortality associated with alternative policy options for primary care and the Mais Médicos (More Doctors) Program in Brazil: forecasting future scenarios

**DOI:** 10.26633/RPSP.2020.31

**Published:** 2020-03-31

**Authors:** Gabriel Vivas Francesconi, Renato Tasca, Sanjay Basu, Thiago Augusto Hernandes Rocha, Davide Rasella

**Affiliations:** 1 Pan American Health Organization/World Health Organization Pan American Health Organization/World Health Organization BrasíliaDistrito Federal Brazil Pan American Health Organization/World Health Organization, Brasília, Distrito Federal, Brazil.; 2 Center for Population Health Sciences School of Medicine, Stanford University StanfordCalifornia United States of America Center for Population Health Sciences, School of Medicine, Stanford University, Stanford, California, United States of America.; 3 Public Health Institute Federal University of Bahia SalvadorBahia Brazil Public Health Institute, Federal University of Bahia, Salvador, Bahia, Brazil.

**Keywords:** Program evaluation, primary health care, computer simulation, mortality, Brazil, Evaluación de programas y proyectos de salud, atención primaria de salud, simulación por computador, mortalidad, Brasil, Avaliação de programas e projetos de saúde, atenção primária à saúde, simulação por computador, mortalidade, Brasil

## Abstract

**Objective.:**

To forecast the impact of alternative scenarios of coverage changes in Brazil’s Family Health Strategy (Estratégia Saúde da Família) (ESF)—due to fiscal austerity measures and to the end of the Mais Médicos (More Doctors) Program (PMM)—on overall under-5 mortality rates (U5MRs) and under-70 mortality rates (U70MRs) from ambulatory care sensitive conditions (ACSCs) up through 2030.

**Methods.:**

A synthetic cohort of 5 507 Brazilian municipalities was created for the period 2017-2030. A municipal-level microsimulation model was developed and validated using longitudinal data. Reductions in ESF coverage, and its effects on U5MRs and U70MRs from ACSCs, were forecast based on two probable austerity scenarios, as compared to the maintenance of current ESF coverage. Fixed effects longitudinal regression models were employed to account for secular trends, demographic and socioeconomic changes, variables related to health care, and program duration effects.

**Results.:**

In comparison to maintaining stable ESF coverage, with the decrease in ESF coverage due to austerity measures and PMM termination, the mean U5MR and U70MR would be 13.2% and 8.6% higher, respectively, in 2030. The end of PMM would be responsible for a mean U5MR from ACSCs that is 4.3% higher and a U70MR from ACSCs that is 2.8% higher in 2030. The reduction of PMM coverage due only to the withdrawal of Cuban doctors who have been working in PMM would alone be responsible for a U5MR that is 3.2% higher, and a U70MR that is 2.0% higher in 2030.

**Conclusions.:**

Reductions in primary health care coverage due to austerity measures and the end of the PMM could be responsible for many avoidable adult and child deaths in coming years in Brazil.

## RESUMEN

**Objetivo.:**

Proyectar el impacto de los distintos escenarios alternativos de cambios en la cobertura de la estrategia de salud familiar de Brasil (Estratégia Saúde da Família o ESF) —motivados por medidas de austeridad y la desaparición del programa *Mais Médicos* (PMM)— en las tasas generales de mortalidad de menores de 5 años y menores de 70 años debidas a trastornos sensibles al cuidado ambulatorio hasta el año 2030.

**Métodos.:**

Se formó una cohorte sintética de 5 507 municipios brasileños para el período 2017-2030. Se elaboró un modelo de microsimulación a escala municipal y se lo validó empleando datos longitudinales. Se proyectaron las reducciones de la cobertura de la ESF y sus efectos sobre la tasa de mortalidad de menores de 5 años y menores de 70 años debida a trastornos sensibles al cuidado ambulatorio con base en dos contextos probables de austeridad, en comparación con el mantenimiento de la actual cobertura de la ESF. Se emplearon modelos de regresión longitudinal con efectos fijos para dar cuenta de las tendencias históricas, los cambios demográficos y socioeconómicos, las variables relacionadas con la atención de salud y los efectos de la duración del programa.

**Resultados.:**

En comparación con el mantenimiento de una cobertura estable de la ESF, ante su disminución por medidas de austeridad y la desaparición del PMM, las tasas medias de mortalidad de menores de 5 años y menores de 70 aumentarían en 13,2 % y 8,6 % respectivamente para el año 2030. La desaparición del PMM sería responsable de una tasa media de mortalidad de menores de 5 años debida a trastornos sensibles al cuidado ambulatorio que será un 4,3 % mayor y, en el caso de los menores de 70 años, un 2,8 % mayor para el año 2030. Tan solo la reducción de la cobertura de PMM exclusivamente a raíz de la retirada de los médicos cubanos que han trabajado en este programa daría cuenta de un incremento del 3,2 % de la tasa de mortalidad de menores de 5 años y del 2,0 % en el caso de los menores de 70 años para el año 2030.

**Conclusiones.:**

Las reducciones de la cobertura de la atención primaria de salud debidas a medidas de austeridad y la desaparición del PMM podrían ser responsables de muchas muertes evitables de niños y adultos en los próximos años en Brasil.

## RESUMO

**Objetivo.:**

Fazer uma projeção da repercussão de cenários alternativos, com a mudança na cobertura da Estratégia de Saúde da Família (ESF) no Brasil decorrente de medidas de austeridade fiscal e do fim do Programa Mais Médicos, nas taxas de mortalidade em crianças menores de 5 anos (TM-5) e taxas de mortalidade em indivíduos até 70 anos (TM-70) por causas sensíveis à atenção ambulatorial até 2030.

**Métodos.:**

Esta análise se baseou em uma coorte sintética de 5.507 municípios brasileiros criada para o período 2017–2030. Um modelo de microssimulação ao nível municipal foi desenvolvido e validado com dados longitudinais. A diminuição da cobertura da ESF e sua repercussão nas TM-5 e TM-70 por causas sensíveis à atenção ambulatorial foram projetadas em dois cenários prováveis de austeridade comparados à continuidade da cobertura atual da ESF. Modelos de regressão com efeitos fixos para dados longitudinais foram usados para levar em consideração as tendências seculares, as variações populacionais e socioeconômicas, as variáveis relacionadas à assistência de saúde e os efeitos da continuidade do programa.

**Resultados.:**

Comparando-se à continuidade da cobertura estável da ESF, com a diminuição da cobertura decorrente de medidas de austeridade e do fim do Programa Mais Médicos, as TM-5 e TM-70 médias seriam 13,2% e 8,6% maiores em 2030. O fim do Programa Mais Médicos resultaria em um aumento de 4,3% na TM-5 média e de 2,8% na TM-70 média por causas sensíveis à atenção ambulatorial em 2030. A diminuição da cobertura do Programa Mais Médicos decorrente exclusivamente da saída dos médicos cubanos do programa estaria associada a um aumento de 3,2% na TM-5 e de 2,0% na TM-70 em 2030.

**Conclusões.:**

A diminuição na cobertura da atenção primária à saúde decorrente de medidas de austeridade e do fim do Programa Mais Médicos teria como resultado muitas mortes evitáveis em adultos e crianças no Brasil nos anos que estão por vir.

The Programa Mais Médicos (More Doctors Program) (PMM) is an initiative of the Brazilian Government whose purpose is to train medical personnel for the country’s Unified Health System (SUS). Legislation establishing the PMM was approved in 2013, with the primary objectives of strengthening SUS services at the primary care level, especially by decreasing the shortage of SUS doctors in underserved regions; reducing health inequalities; and improving medical education in the country (1). An important aspect of this program was the possibility of placing new doctors into primary health care according to the criteria of equity, social vulnerability, and specific populations (2, 3). In order to do this, the program invested in strategies for attracting, training, and retaining physicians; improving the infrastructure of basic health units; and increasing the number of places in medical school and of primary care residencies (4).

The PMM aims to provide physicians to disadvantaged areas that lack medical professionals. Using a recruitment approach supported by an international cooperation effort, the program mobilized nearly 20 000 medical professionals and filled more than 11 400 PMM positions. A total of 4 058 municipalities benefited, as well as 100% of the country’s Special Indigenous Health Districts (DSEIs) (5). In regions where there was a shortage of professionals, the offer of jobs in the PMM was primarily aimed at Brazilian doctors. Subsequently, vacancies not occupied by Brazilian professionals were made available to professionals from other countries, through a cooperative effort by the Government of Brazil, the Government of Cuba, and the Pan American Health Organization. The PMM has helped reduce the shortage of doctors in SUS priority areas, strengthen primary care services, and increase access to and the quality of basic care services (4).

After almost five years of implementation, the PMM has added both strategic value in reducing gaps in health equality and has capitalized on the unique nature of the Cuba-Brazil South-South cooperation experience, triangulated through the Pan American Health Organization/World Health Organization (PAHO/WHO) (3). According to PAHO, 8 500 Cuban doctors were working in Brazil’s Estratégia Saúde da Família (Family Health Strategy) (ESF) and in the Indigenous Health Strategy. The Cuban doctors were responsible for providing basic health service to about 8 million families (almost 30 million individuals) in 2 800 municipalities (6). In November 2018, the withdrawal of Cuban doctors from the PMM fostered a discussion among policymakers regarding what might be the impact on the Brazilian health system. Millions of Brazilians, most of whom live in remote, vulnerable areas, could be left without health care after Cuban authorities announced that their country was withdrawing its doctors from the PMM (6).

Taking into consideration these new challenges presented for the Brazilian primary care initiatives, the main objective of this study is to analyze possible scenarios regarding the termination of the PMM. We aim to rigorously forecast potential health impacts of alternative ESF coverage scenarios through 2030. The forecasting effort was made using a microsimulation approach.

## METHODS

We created a cohort of 5 507 municipalities for the years 2017-2030 using municipal-level discrete-time microsimulation modeling based on the extension of a longitudinal data set of the same municipalities for the years 2000-2016, which had been used in previous ESF impact evaluations (7, 8). The use of such an approach addresses many of the limitations of deterministic cohort models because the microsimulation model can more accurately reflect individual clinical pathways, incorporate the impact of history on future events, and more easily capture the variation in patients’ characteristics at baseline (9).

The microsimulation was developed starting from two already validated microsimulation models. The first model considered the under-5 mortality rate (U5MR) due to ambulatory care sensitive conditions (ACSCs) as the outcome. The second model addressed the under-70 mortality rate (U70MR), also from ACSCs. Both models were recently employed in forecasting studies regarding the effects of socioeconomic changes in Brazil (10). The U5MR and U70MR microsimulation models were built from data and parameters of already published retrospective analyses (10). The earlier analyses used a longitudinal fixed effects regression approach (11). The regressions fitted were adjusted for municipal-level demographic and socioeconomic factors. By using the designed approach, it was possible to predict changes in future U5MRs and U70MRs due to ACSCs, based on known associations with changes in primary care coverage.

The modeling approach was undertaken in two stages. The first stage was to generate a synthetic cohort of municipalities with municipal-level forecast values. The data from 2000-2016 was used to build a synthetic cohort covering from 2013 to 2030. The variables considered in this process were related to socio-economic status, health system characteristics, the ESF, and the Family Allowance Program (PBF). The second modeling stage consisted of predicting U5MRs and U70MRs from the ACSCs, based on all these variables, beginning with 2010. The data from the years before 2017 were used to calibrate and validate the model. (Further details on the microsimulation models can be requested from the authors, including on the modeling calibration process, internal and external validation, and model equations in accordance with the international model reporting guidelines (ISPOR-SMDM) (10)).

Considering the possibilities of applying such an approach in the health domain, we forecast four future scenarios, representing a range of primary care policy options in Brazil.

The first was a status quo scenario, where municipal ESF coverage (mean municipal coverage of 80.4% in 2016) remains the same. This scenario assumes no deterioration in ESF effectiveness in health outcomes, such as from reducing services or the quality provided while maintaining coverage figures.

The second scenario, of contracting ESF coverage, related to the effects of a reduction in federal health care spending, where municipal ESF coverage declines proportionally to the federal reductions in per capita health expenditure as defined by the country’s Constitutional Amendment 95 (EC 95) (12). These declines in ESF coverage are based on published forecasts (13), while a range of possible declines have been tested in sensitivity analyses.

The third scenario considered the contraction of the ESF coverage, as above, but with also the complete termination of the PMM. This scenario models specific ESF coverage declines in municipalities where the PMM operates.

The fourth scenario estimated the impact of the contraction of ESF coverage as under Scenario 1, plus the complete withdrawal of the Cuban doctors in the PMM.

### Data sources

Two data sets were used for input parameters, supplemented with additional data sources. The first data set contained annual municipal demographic, socioeconomic, social assistance, and health system variables. These data were obtained from publicly available sources, including the websites of the Brazilian Ministry of Health (DATASUS) (14) and the Brazilian Institute of Geography and Statistics (IBGE) (15, 16). Socioeconomic variables were forecast from 2011 forward through exponential decay formulas using historical trends of the previous decade (2000-2010) for each municipality and each variable, and, in the case of the poverty rate, calibrated with national estimates from the National Household Surveys for the period 2011-2014 (16). Poverty rates for each municipality from 2015 (during an economic recession) were calibrated according to World Bank estimates, as described elsewhere (17). Annual municipal ESF coverage and PBF coverage values up through 2016 were obtained from the Brazilian Ministry of Health’s Department of Primary Care (18) and Brazilian Ministry of Social Development (19). The data was also supplemented with information on the number of PMM professionals in Brazilian municipalities, obtained from the PAHO/WHO Brazil Country Office. The density of PMM professionals in each municipality, overall and for only Cuban doctors, was used to calculate the percentage of the primary care coverage carried out under the PMM.

The second data set contained effect sizes of the associations between changes in the primary care coverage and sociodemographic variables, as well as changes in municipal U5MRs and U70MRs from ACSCs. The effect sizes were all obtained from supplementary web materials published with earlier articles (8, 20, 21) with longitudinal fixed effects multiple regression models.

### Simulation of the effects on mortality

Using all the independent variable values from the retrospective synthetic cohort of municipalities, we performed a microsimulation forecasting analysis. The estimation approach was carried out using the longitudinal fixed effects multiple regression model of mortality rates. This made it possible to assess the retrospective impact evaluations (8), starting from 2010, in order to calibrate and validate the model. For each out-come and each of the four ESF scenarios, 10 000 Monte Carlo simulations were performed. This allowed the parameters to vary according to their underlying distributions. Taking into consideration the data characteristics, a Poisson distribution was used for baseline mortality rates, and normal distributions for all other variables and parameters. For the effect sizes of each variable, the variance of the normal distribution was calibrated with the confidence interval of its effect size parameter (rate ratios) from reference studies (8, 22). Mean values and credible intervals (CrIs) (the 2.5% and 97.5% quantiles of the distribution) are reported.

### Calibration and validation of the models

As all model parameters were derived from real data and estimates of the retrospective impact evaluations for U5MRs and U70MRs from ACSCs, the only necessary calibration was of the secular trends (assuming it would have been different from the previous decade). Internal validity of the model was assessed by fitting the same fixed effects longitudinal regression models employed in the retrospective impact evaluations. To validate the model fitted through the microsimulation forecasting approach, the same approach was used. This made it possible to assess the validity of the findings on the synthetic dataset produced by the microsimulation. After the validation process the obtained coefficients were identical to those introduced as inputs in the model. External validation of the model was undertaken for U5MRs by comparing the overall national U5MR forecast by our microsimulation with the official Brazilian U5MR estimates (14). For the external validation of U5MRs from ACSCs, data from the Global Burden of Disease (http://www.healthdata.org/gbd) were used. The proportion of variance (R^2^) explained was also examined, and the observed values were verified to ensure they fell within the simulated 95% CrIs. (Further details on model calibration and validation are available from the authors.) The model was coded and implemented in R version 3.4.0 programming language.

### Sensitivity analysis

Multiple sensitivity analyses were undertaken to verify model robustness and assumptions within the ESF scenarios. First, the potential effect from different economic crises and changes in poverty rates were tested, according to a forecast from previous research (8, 21). Second, alternative situations of contracting ESF coverage were tested to model the possibility of a higher and lesser impact of austerity cuts on ESF coverage. Third, longer ESF coverage duration effects were explored, and the ESF effect size was also stratified based on the poverty rate level of the municipalities. Finally, different time trends were evaluated to test the impact of different approaches to modeling secular trends in U5MRs and U70MRs from ACSCs.

## RESULTS

Table 1 shows the mean values of municipality ESF coverage and values of the covariates employed in the models. As modeled under Scenario 1 (status quo), the mean of municipality ESF coverage would remain constant (at 80.4%) until 2030. Under Scenario 2 (contracting ESF coverage due to a reduction of federal health care spending) and Scenario 3 (contracting ESF coverage and PMM termination), mean coverages would fall to 37.8% and 16.0%, respectively. Under Scenario 4 (contracting ESF coverage and the withdrawal of the Cuban doctors), mean coverage would fall from 84.7% in 2015 to 22.0% in 2030.

**FIGURE 1.**
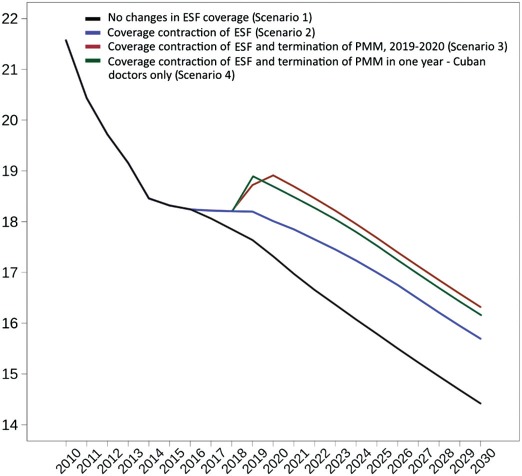
Mean municipal under-5 mortality rates (per 1 000 live births) from ambulatory care sensitive conditions, under four scenarios for coverage changes in Brazil’s Family Health Strategy (ESF), due to fiscal austerity measures and to the end of the Mais Médicos Program (PMM), 2010-2030

***Source:*** Prepared by the authors, based on the study results.

**FIGURE 2.**
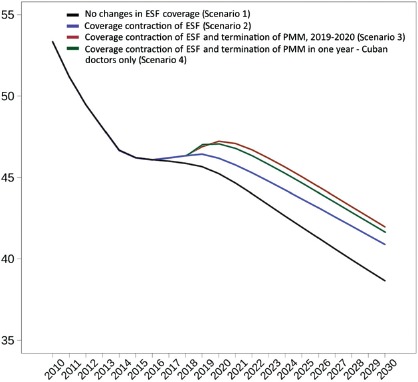
Mean municipal under-70 mortality rates (per 100 000) from ambulatory care sensitive conditions, under four scenarios for coverage changes in Brazil’s Family Health Strategy (ESF), due to fiscal austerity measures and to the end of the Mais Médicos Program (PMM), 2010-2030

***Source:*** Prepared by the authors, based on the study results.

Under all four scenarios, mean municipal U5MRs and U70MRs from ACSCs (Figure 1 and Figure 2, respectively) are forecast to continue declining, albeit at different rates and with some yearly increases, depending on the specific scenario.

With the decrease in ESF coverage due to the reduction in federal health care spending and PMM termination (Scenario 3), in comparison to maintaining stable ESF coverage, the mean U5MR would be 13.2% higher in 2030 (95% CrI: 11.7%-14.7%), with 43 732 excess under-5 deaths for 2017-2030. The end of PMM itself would be responsible for a mean U5MR 4.3% higher (95% CrI: 2.6%-5.4%), with 15 198 excess under-5 deaths in 2030 (comparing Scenario 2 and Scenario 3). The reduction of PMM coverage due only to the withdrawal (and no replacement) of Cuban doctors would be responsible for a U5MR 3.2% higher (95% CrI: 1.6%-4.3%), with 10 428 excess under-5 deaths, in 2030 (comparing Scenario 2 and Scenario 4) (Table 2).

Concerning U70MRs from ACSCs, with the decrease in ESF coverage due to the reduction in federal health care spending and the PMM termination, mean municipal mortality would be 8.6% higher in 2030 (95% CrI: 7.1%-10.2%), with 48 232 excess deaths. Considering the end of the PMM, the U70MR from ACSCs would be 2.8% higher (95% CrI: 1.1%-4.2%), with 20 457 excess under-70 deaths from ACSCs, in 2030 (comparing Sce-nario 2 and Scenario 3). The reduction in PMM coverage due only to the withdrawal (and no replacement) of Cuban doctors would be responsible for the U70MR from ACSCs being 2.0% higher (95% CrI: 0.4%-3.4%) in 2030, with 12 412 excess under-70 deaths from ACSCs (comparing Scenario 3 and Scenario 4) (Table 3).

**TABLE 1.** Mean value and standard deviation (SD) of coverage levels in Brazil’s Family Health Strategy (ESF) for the years 2015, 2020, and 2030, in study of mortality associated with alternative policy options for primary care and the Mais Médicos (More Doctors) Program (PMM) in Brazil

Scenario(s)^a^	Variable	2015 (mean and SD)	2020 (mean and SD)	2030 (mean and SD)
Static ESF coverage (Scenario 1)	ESF coverage	84.7% (22.2%)	80.4% (23.5%)	80.4% (23.5%)
Contracting ESF coverage (Scenario 2)	ESF coverage	84.7% (22.2%)	66.5% (20.0%)	37.8% (13.9%)
Contracting ESF coverage and PMM termination (Scenario 3)	ESF coverage	84.7% (22.2%)	37.0% (30.0%)	16.0% (18.6%)
Contracting ESF coverage and withdrawal of Cuban Doctors from the PMM (Scenario 4)	ESF coverage	84.7% (22.2%)	44.9% (29.2%)	22.0% (16.7%)
All scenarios	PBF municipal coverage	33.5% (22.1%)	37.9% (29.1%)	29.7% (25.3%)
	Poverty rate	13.8 % (15.3%)	18.4 % (21.5%)	12.0 % (17.4%)
	Log of illiteracy rate	-2.37 (0.79)	-2.98 (1.06)	-3.38 (1.27)
	Urbanization rate	68.4% (22.2%)	71.5% (22.4%)	75.8% (23.1%)
	Public hospital beds	0.13 (0.21)	0.12 (0.29)	0.13 (0.25)
	Private hospital beds	0.04 (0.10)	0.05 (0.11)	0.05 (0.12)
	Log of private health care insurance	-5.62 (1.62)	-5.52 (1.66)	-5.46 (1.73)
	Log of GDP per capita	-4.70 (0.71)	-4.54 (1.02)	-4.48 (1.46)

***Source:*** Prepared by the authors, based on the study results.

a For all the scenarios, ESF coverage, Family Allowance Program (PBF) coverage, poverty rate, and urbanization rate are expressed as percentages. In addition, private health care insurance and gross domestic product (GDP) per capita are log-transformed; the illiteracy rate is for those aged 25 yr and over and is log-transformed; and public and private hospital beds are expressed per 1 000 municipality inhabitants.

### Sensitivity analysis

Findings from our sensitivity analyses support the robustness of the findings. Firstly, varying the magnitude of declines in municipal ESF coverage associated with reduction in federal health care spending reveals that under a range of values of mortality impacts, there is a dose-response relationship (regarding deeper ESF contractions and smaller reductions in ACSC mortality) that is consistent with the primary analyses. Introducing different duration effects of the ESF coverage produced similar results, as did varying the effectiveness of ESF in reducing ACSC mortality between municipalities with different levels of poverty. Modeling different economic recessions and changes in poverty rates over the coming years yielded highly similar results, both in aggregate and inequality analyses. Intro-ducing different secular trends in U5MRs and U70MRs from ACSCs alters the general trends over time, but the relative dif-ferences between scenarios (regarding the rate ratios) remain unchanged.

**TABLE 2.** Ratio of under-5 mortality rates (U5MRs) from ambulatory care sensitive conditions (ACSCs) in 2020 and 2030 in Brazil’s Family Health Strategy (ESF), as compared to constant ESF coverage, in study of mortality associated with alternative policy options for primary care and the Mais Médicos (More Doctors) Program (PMM) in Brazil

Scenario	2020		2030	
	Ratio of ACSC mortality rates	Credible interval	Ratio of ACSC mortality rates	Credible interval
Static ESF coverage (Scenario 1)	1 (reference)	NA^a^	1 (reference)	NA
Contracting ESF coverage (Scenario 2)	1.040	1.026-1.054	1.089	1.074-1.103
Contracting ESF coverage and PMM termination (Scenario 3)	1.092	1.077-1.107	1.132	1.117-1.147
Contracting ESF coverage and the withdrawal of the Cuban doctors from the PMM (Scenario 4)	1.079	1.065-1.094	1.121	1.106-1.136

***Source:*** Prepared by the authors, based on the study results.

a NA = not applicable.

**TABLE 3.** Ratios of under-70 mortality rates (U70MRs) from ambulatory care sensitive conditions (ACSCs) under Family Health Strategy (ESF) scenarios, compared to constant ESF coverage in 2020 and 2030, in study of mortality associated with alternative policy options for primary care and the Mais Médicos (More Doctors) Program (PMM) in Brazil

Scenario	2020		2030	
	Ratio of ACSC mortality rates	Credible interval	Ratio of ACSC mortality rates	Credible interval
Static ESF coverage (Scenario 1)	1 (reference)	NA^a^	1 (reference)	NA^a^
Contracting ESF coverage (Scenario 2)	1.021	1.006-1.036	1.058	1.042-1.074
Contracting ESF coverage and PMM termination (Scenario 3)	1.044	1.029-1.059	1.086	1.071-1.102
Contracting ESF coverage and the withdrawal of the Cuban doctors from the PMM (Scenario 4)	1.041	1.026-1.056	1.078	1.063-1.094

***Source:*** Prepared by the authors, based on the study results.

a NA = not applicable.

## DISCUSSION

The primary objective of this research was to investigate how different perspectives concerning ESF coverage might be related to U5MRs and U70MRs from ACSCs. The four scenarios investigated reflect the current changes in the Brazilian context regarding a political, economic, and health care point of view. Our estimates suggest that reductions in ESF coverage, due to reductions in federal health care expenditures and the end of the PMM (or, at least, the nonreplacement of the Cuban doctors in the PMM) could be responsible for a large number of child and adult deaths through 2030, reaching the toll of almost 100 000 premature deaths over the period. This number is still an underestimate of the overall impact of ESF reductions in coverage. That is because U70 deaths represent around 55% of all Brazilian deaths, U70 deaths from ACSCs are only around 13% of all U70 deaths in Brazil, and ESF has effects (although probably smaller in magnitude) in other mortality causes (23).

Since 2015, an economic crisis has imposed a series of challenges to managing health care policies in Brazil (17). The reduction in the gross domestic product (GDP) was associated with increases in unemployment and the poverty rate (17). To face this crisis, in 2016 the National Congress approved a constitutional amendment (EC 95). It imposed a limit for all federal government expenditures, except payment of public debt, defined by the inflation rate of the previous year, for the next 20 years (13, 17). The potential impact of EC 95 on the health system in coming years has been explored in several studies (13, 17, 24). The probability of a contraction in ESF investments is strong, especially because the freezing of health care spending due to EC 95 has come along with the new National Primary Health Care Policy (PNAB). The new PNAB exempts Brazilian states and municipalities from applying the transferred federal funds to the ESF (25). Additionally, Brazil is currently facing the need to restructure the PMM, given the changes resulting from Cuba’s withdrawal from the technical cooperation with PAHO/WHO (6). The limitation of public expenditure on health, in combination with a restructuring of the PMM, has produced a discussion among policymakers regarding the consequences of such measures in terms of population health.

The findings of this study highlight a challenging scenario. The repatriation of the Cuban doctors has threatened primary care services for nearly 30 million people (6). The migration of doctors from ESF teams to the PMM vacancies is especially worrying. For several years, the competition for doctors has been part of the daily challenges faced by municipal health secretariats in Brazil (28). The municipalities cannot compete with the federal government regarding salary conditions due to restrictions imposed by a Brazilian law of fiscal responsibility (27) that sets an upper limit on salaries for each level of public administration.

The PMM is the initiative that has had the best results in terms of attracting physicians to remote and disadvantaged areas and keeping them there (28). An abrupt change in the logic of the program might have consequences for the health needs of the population. The estimates obtained in our research highlight the urgency of new approaches capable of handling the withdrawal of the Cuban doctors. The formulation of new policies should take into consideration the lessons from the past concerning effective strategies capable of attracting and keeping health professionals where they are needed. Without guaranteed access to adequate primary care services for deprived populations, it will be hard to ensure the accomplishment of the universal health coverage objective (29).

The recent changes in the Brazilian circumstances have launched the country into a double challenge. Cuts in health care spending have come on top of old challenges associated with inequity in the distribution of the medical work force. The potential excess deaths projected in our research may be associated with other health problems, such as additional costs for emergency services (30, 31) and an increase in disability-adjusted life years (32).

Brazil is currently affected by a triple burden of diseases: an unsolved agenda of injuries, high rates of infectious diseases, and an increasing burden of noncommunicable conditions resulting from the epidemiological transition. The best approach to handle noncommunicable conditions is based on strong primary care (33). It is quite challenging for a health system to effectively manage the increasing costs associated with a high burden of noncommunicable diseases without the support of primary care. The decrease in the ESF coverage would be associated with an increase in U5MRs and U70MRs due to conditions dependent on ambulatory care.

The difficulty in readily replacing the Cuban doctors could contribute to the results projected by the simulations we have performed. To avoid the negative consequences forecast through our work, a monitoring effort will be needed to identify necessary strategic changes. In December of 2018, Brazil had 39 872 ESF teams. The 8 471 Cuban doctors were responsible for approximately 21% of the primary care coverage in Brazil (6). Without a prompt, effective response by the Brazilian Ministry of Health, almost one-quarter of the primary care services of the country may be affected by the shortage of professionals. The announcements seeking Brazilian doctors to replace the Cuban work positions filled all the remaining vacancies. However, in some instances, there have been high turnover rates among the recently hired doctors, with some of them leaving in just a month after starting.

This study does not consider the effect of new policies that may be implemented in Brazil. The formulation of initiatives capable of addressing the inequity in the distribution of doctors may help minimize the possible harms we have projected. Despite this, the microsimulation approach was robust in terms of sensitivity concerning the findings. Our microsimulation approach used Monte Carlo methods (9). This methodology relies on repeated random sampling to obtain numerical results, using randomness to solve a problem that might be deterministic in principle.

To overcome the current scenario, the Brazilian health system will require innovative, strategic approaches. The efforts chosen will need to be carried out in a context of limited resources and high demand for effective interventions. The policy discussion will need evidence capable of supporting decisions and circumventing obstacles faced in the past. Otherwise, the scarce resources available could be misspent.

### Authors contributions.

GVF, RT, TAHR, and DR participated in the design of the study, interpretation of the data, and writing of the text. All authors reviewed and approved the final version.

### Financial support.

This study received funding from the Wellcome Trust Training Fellowships in Public Health and Tropical Medicine scheme (with DR as the recipient Fellow (grant reference number 109949/Z/15/Z; https://wellcome.ac.uk/)) and from the Pan American Health Organization (consultancy contract CON18-00025710). Neither of the two funders had any role in the study design, data collection, analysis and modeling, interpretation of the results, or writing of the manuscript.

### Disclaimer.

The authors hold sole responsibility for the views expressed in the manuscript, which may not necessarily reflect the opinion or policy of the *RPSP/PAJPH* and/or PAHO.
